# Radio-Frequency-Controlled Urea Dosing for NH_3_-SCR Catalysts: NH_3_ Storage Influence to Catalyst Performance under Transient Conditions

**DOI:** 10.3390/s17122746

**Published:** 2017-11-28

**Authors:** Markus Dietrich, Gunter Hagen, Willibald Reitmeier, Katharina Burger, Markus Hien, Philippe Grass, David Kubinski, Jaco Visser, Ralf Moos

**Affiliations:** 1 Bayreuth Engine Research Center (BERC), Department of Functional Materials, University of Bayreuth, 95447 Bayreuth, Germany; Markus.Dietrich@continental-corporation.com (M.D.); functional.materials@uni-bayreuth.de (G.H.); 2 Continental Automotive GmbH, Division Powertrain, 93055 Regensburg, Germany; Willibald.Reitmeier@continental-corporation.com (W.R.); Katharina.2.Burger@continental-corporation.com (K.B.); Markus.Hien@continental-corporation.com (M.H.); Philippe.Grass@continental-corporation.com (P.G.); 3 Ford Research and Innovation Center, Dearborn, MI 48124, USA; dkubinsk@ford.com (D.K.); jvisser@ford.com (J.V.)

**Keywords:** radio-frequency (RF), NH_3_ SCR, NH_3_ storage, NO_x_ conversion, NH_3_ slip, direct control, microwave cavity perturbation, transient conditions, storage influence

## Abstract

Current developments in exhaust gas aftertreatment led to a huge mistrust in diesel driven passenger cars due to their NO_x_ emissions being too high. The selective catalytic reduction (SCR) with ammonia (NH_3_) as reducing agent is the only approach today with the capability to meet upcoming emission limits. Therefore, the radio-frequency-based (RF) catalyst state determination to monitor the NH_3_ loading on SCR catalysts has a huge potential in emission reduction. Recent work on this topic proved the basic capability of this technique under realistic conditions on an engine test bench. In these studies, an RF system calibration for the serial type SCR catalyst Cu-SSZ-13 was developed and different approaches for a temperature dependent NH_3_ storage were determined. This paper continues this work and uses a fully calibrated RF-SCR system under transient conditions to compare different directly measured and controlled NH_3_ storage levels, and NH_3_ target curves. It could be clearly demonstrated that the right NH_3_ target curve, together with a direct control on the desired level by the RF system, is able to operate the SCR system with the maximum possible NO_x_ conversion efficiency and without NH_3_ slip.

## 1. Introduction

Since 2015, the exhaust gas aftertreatment for diesel engine driven vehicles has been of particular interest in the realm of politics, society, and media. After it became public that many cars with ammonia-based selective catalytic reduction (NH_3_ SCR) catalysts use illegal shut-off devices of the aftertreatment system, there is a huge mistrust in diesel driven passenger cars. However, the NH_3_-SCR is the only current DeNO_x_ strategy for light and heavy-duty diesel engines with the capability to meet the current and upcoming emission legislations [1,2,3].

In this technique, aqueous urea solution of 32.5 wt % in water (diesel exhaust fluid = DEF, AdBlue^TM^, or AUS32 = aqueous urea solution) is injected into the exhaust to reduce toxic nitric oxides (NO_x_ = NO + NO_2_) on special SCR catalysts. After injection into the hot exhaust (>180 °C), the urea solution decomposes after water evaporation by thermolysis and hydrolysis into gaseous ammonia (NH_3_) and carbon dioxide (CO_2_). The formed ammonia adsorbs on the active sites of the SCR catalyst and is able to reduce NO_x_ into nitrogen (N_2_) and water (H_2_O) [2,4]. Owing to the present reaction pathways, the previous NH_3_ adsorption is the essential precondition for all SCR reactions [5,6,7,8]. The two most important SCR reactions are the so-called standard SCR reaction (Equation (1)) and the fast SCR reaction (Equation (2)), which occur depending on the present NO/NO_2_ ratio. Whereas in the standard SCR oxygen (O_2_) participates, the fast SCR reaction requires an equimolar amount of NO, NO_2_, and no O_2_ [2,4].

4 NH_3_ + 4 NO + O_2_ → 4 N_2_ + 6 H_2_O (1)

4 NH_3_ + 2 NO + 2 NO_2_ → 4 N_2_ + 6 H_2_O (2)

The required previous NH_3_ adsorption also offers additional benefits for the NO_x_ conversion efficiency and the SCR system control. Due to the kinetical limitations of the SCR reactions, a specific NH_3_ surface coverage on the catalyst is required to achieve high NO_x_ conversion, especially in the lower temperature range (<300 °C) [8,9]. Additionally, this leads to an easier dosing control, since changes in NO_x_ emissions and gas flow from transient driving conditions can be buffered. Therefore, the catalyst material development aims for SCR catalysts with high NH_3_ storage capacity and high activity at low temperatures [3]. The latter is also required due to improvements in diesel engine technology, leading to continuously decreasing exhaust gas temperatures. The current SCR catalysts are mostly based on copper (Cu) exchanged zeolites that combine the above-mentioned requirements [10,11,12,13].

Today’s SCR system or DEF dosing control is model-based and uses gas sensor signals (NO_x_ and/or NH_3_ sensors), and other data from the engine control unit, like the calculated exhaust gas mass flow, to approximate the actual amount of stored NH_3_ on the catalyst surface [14]. The applied models simulate the entire NH_3_ ad- and desorption equilibrium and all occurring reactions on the catalyst, and calculate the required NH_3_ to control the DEF dosing module [15,16,17]. This approach, however, suffers from the fact that already-existing small errors or deviations of only one part of the whole control system may lead to an over- or under-estimation of the actual amount of stored NH_3_, followed by NO_x_ or NH_3_ emissions [18,19].

This paper reports on the continuation of the development of a radio-frequency-based (RF) measurement technique as the only approach to monitor directly (*in operando*) the current amount of stored NH_3_ on SCR catalysts. Therefore, it relies on our recently published paper [20] and uses the developed system calibration for a first transient study with automatic DEF dosing control only relying on the RF system.

## 2. Radio-Frequency-Based (RF) Catalyst State Monitoring

In the RF- or microwave-based catalyst or filter state determination, the catalyst or filter itself operates as the sensitive part of the sensor system. By coupling electromagnetic waves into the metal canning, resonances (i.e., standing electromagnetic waves) can be excited at specific frequencies. The resonance frequencies are dependent on the canning’s geometry and dielectric properties of the catalyst or filter. The detectable state of the catalyst or filter requires a change of the dielectric properties of the material related to its state and can be detected directly by analyzing the resonance parameters [21,22,23].

The functionality of this approach has been proven for several types of automotive catalyst and filter systems, starting with the oxidation state of three-way catalytic converters (TWC) related to the conductivity of oxidized or reduced Ceria as oxygen storage component [24,25,26]. Soot loading on particulate filters for diesel (DPF) or gasoline engines (GPF) can also be monitored related to the conductivity of the accumulated soot particles [27,28,29,30,31]. This technique also might allow a differentiation of soot and ash loading [32]. A combined system with a GPF with TWC functionality was under investigation within transient operation in the European Driving Cycle (NEDC) as well [27]. The detection of NO_x_ storage on lean NO_x_ traps (LNT) is also possible, but the observed catalyst samples showed a comparably small signal and might require further investigation with current catalyst formulations [33,34]. The basic capability of the RF approach to detect the NH_3_ storage on SCR catalysts has been demonstrated for vanadia- and several types of zeolite-based catalysts in previous work [35,36,37,38]. In our most recent work, we performed the step in engine dynamometer setup using DEF, instead of gaseous NH_3_, and proved the functionality of the RF approach for commercial zeolite-based SCR catalysts (Fe and Cu exchanged) under realistic conditions [20,39]. 

The expected measurable material effects related to NH_3_ storage on SCR catalysts are the polar nature of the NH_3_ molecule and its effects to the conductivity mechanisms inside the zeolite structure, due to adsorption on the acidic storage sites [40,41]. Both effects are mirrored in the complex dielectric permittivity (*ε* = *ε*_1_ − j*ε*_2_), and experiments with zeolite-based catalyst powder samples in a special setup proved the linear response of both *ε*_1_ and *ε*_2_ to NH_3_ storage [42,43]. 

If the RF measurement is performed with only one coupling element (e.g., a coaxial probe feed), only one reflection signal can be analyzed with resonances appearing as local minima. If two coupling elements are applied, the number of possible RF signals increases to four, with two reflection and two transmission signals, whereas resonances appear as local maxima in transmission mode. From both types of measurement signals, two resonance parameters can be extracted from each resonance peak: the resonance frequency *f*_res_ and the unloaded quality factor *Q*_0_. Described by the theory of the so-called cavity perturbation method, changes of the resonance frequency Δ*f*_res_/*f*_0_ are related to the changes of the dielectric permittivity Δ*ε*_1_, which is a measure of polarization effects (Equation (3)). Additionally, changes of dielectric losses, including conductivity mechanisms inside the zeolite structure, are represented in Δ*ε*_2_ and mirrored in the change of the reciprocal unloaded quality factor Δ*Q*_0_^−1^ (Equation (4)).

Δ*f*_res_/*f*_0_ ∝ Δ*ε*_1_ (3)

Δ*Q*_0_^−1^ ∝ Δ*ε*_2_ (4)

For more detailed descriptions and theoretical background of the applied cavity perturbation method, including the used assumptions for the application case and determination of the two resonance parameters *f*_res_ and *Q*_0_, we refer to our previous work [27,41,44].

## 3. Experimental Section

### 3.1. Dynamometer Setup

**Figure 1:**
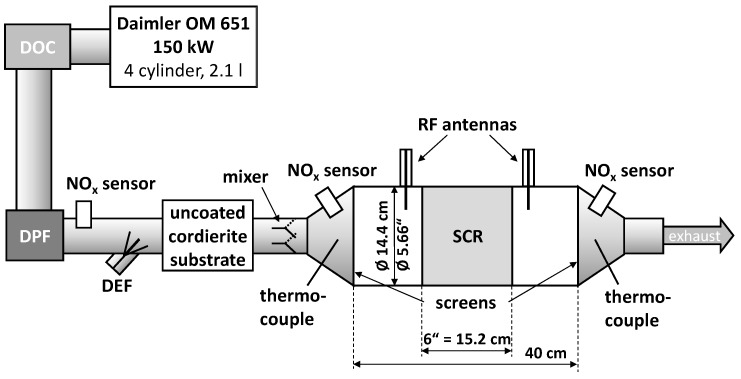
Illustration of the dynamometer setup: 2.1 ltr diesel engine with diesel oxidation catalyst and particulate filter, DEF dosing with uncoated cordierite substrate and plate mixer, Ø 5.66” (Ø 14.2 cm) selective catalytic reduction (SCR) catalyst canning defined by metal screens with two radio-frequency (RF) antennas, thermocouples up- and downstream of the SCR and three NO_x_ sensors up- and downstream of SCR and upstream of DEF dosing, from [20].

As this work directly continues the results of [20], the presented study was performed on the identical engine dynamometer setup and catalyst sample, as illustrated in Figure 1. It is described as follows in the order of gas flow: the turbocharged 4-cylinder and 2.1 liter diesel engine (Daimler OM 651, 150 kW), the serial type diesel oxidation catalyst (DOC) and DPF, the first NO_x_ sensor detecting the raw NO_x_ emissions, the DEF dosing module (Bosch Denoxtronic 3.2), an uncoated cordierite substrate to support NH_3_ formation, a plate mixer for a uniform NH_3_ concentration, the second NO_x_ sensor that detects NO_x_ and, due to its cross sensitivity, also NH_3_, the SCR catalyst canning (Ø 5.66” = 14.4 cm, length 6” = 15.2 cm) and a third NO_x_ sensor to interpret the catalyst conversion efficiency. The catalyst sample is the well-studied copper-exchanged zeolite Cu-SSZ-13 [35,36,45] (Ford Motor Company, Dearborn, MI, USA) and was placed in the middle of the RF canning with two RF antennas, one located up- and one downstream. The ideal cylindrical shape of the resonator is defined by two metal screens and the catalyst temperature is determined indirectly by two thermocouples, located outside of the resonance cavity.

The RF signals were acquired with a vector network analyzer (VNA, MS46322A, Anritsu, Morgan Hill, CA, USA), connected by 50 Ω coaxial cables to the antennas (both not shown in Figure 1). The analyzed resonance is the lowest appearing resonance mode (TE_111_) with one electrical field maximum in the center of the cavity and a linear sensitivity to NH_3_ storage [20,39]. The RF data is measured in transmission mode (complex scattering parameter *S*_21_) and analyzed in a complex manner to determine the two RF parameters *f*_res_ and *Q*_0_. Due to the applied settings, an acquisition rate of 1 Hz was used. A detailed description of the used RF data analysis can be found in [41].

### 3.2. RF System Calibration, NH_3_ Storage Target Curves and Control Flow

**Figure 2:**
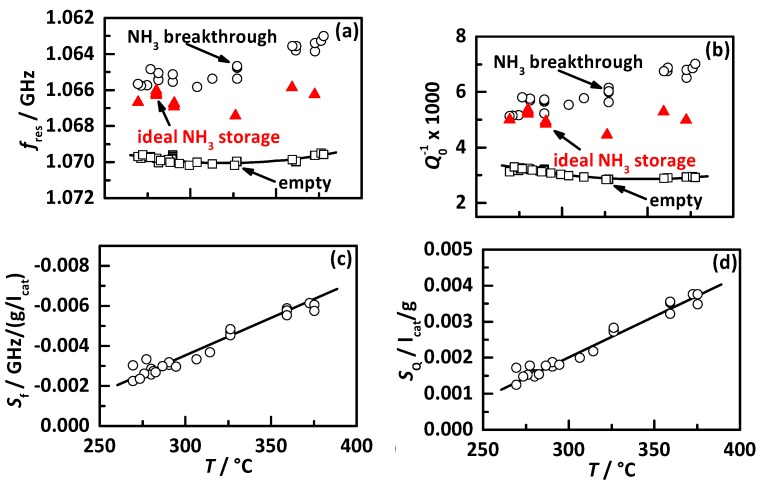
Results of Ref. [20] used for RF system calibration: the RF signals for different states in (**a**) for *f*_res_ and (**b**) for *Q*_0_^−1^ with the empty state (squares), the ideal NH_3_ storage (red triangles) and the NH_3_ loading as first breakthrough occurs (circles), and the determined sensitivities to NH_3_ storage in (**c**) for *f*_res_ and in (**d**) for *Q*_0_^−1^. The used calibration functions for the empty state and the NH_3_ sensitivity for both RF parameters are displayed by black curves.

The determined behavior of the RF signals of [20] is summarized in Figure 2, with *f*_res_ in (a) and *Q*_0_^−1^ in (b) for the empty state (squares). The ideal NH_3_ storage degree (red triangles) represents the minimum NH_3_ storage to achieve the maximum possible NO_x_ conversion efficiency and the NH_3_ storage level when first NH_3_ breakthrough occurs (circles). The determined linear sensitivities to NH_3_ storage of both RF signals are shown in (c), with *S*_f_ for *f*_res_ (Equation (5)), and in (d) with *S*_Q_ for *Q*_0_^−1^ (Equation (6)).

*S*_f_ = Δ*f*_res_/Δ*m*_NH3_ (5)

*S_Q_* = Δ*Q*_0_^−1^/Δ*m*_NH3_ (6)

The used calibration of the RF system to determine the current NH_3_ storage in real time by the RF signals and the catalyst temperature requires the empty state and the NH_3_ sensitivity. They are plotted as solid black curves in Figure 2.

**Figure 3:**
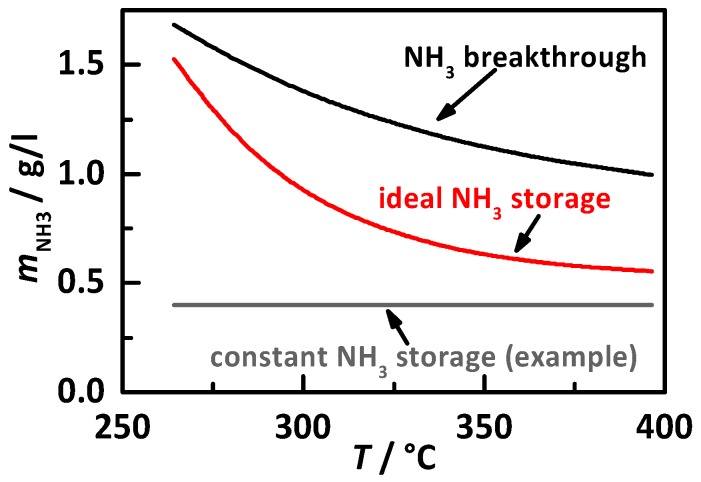
Illustration of the investigated approaches for NH_3_ target storage with percentage gradation of the storage degree when first NH_3_ breakthrough occurs (black) and of the ideal NH_3_ storage curve (red, minimum storage for maximum NO_x_ conversion), both determined in stationary experiments in Ref. [20], and different constant storage levels between 0.2 and 1.4 g/l_cat_ in a gradation of 0.2 g/l_cat_ (example of 0.4 g/l_cat_ displayed in grey).

Besides the RF calibration to determine the current NH_3_ storage on the catalyst surface, the desired NH_3_ storage degree as a function of catalyst temperature is the second necessary part to demonstrate the functionality and possible benefits of an RF-controlled SCR system. Therefore, this study investigates three different approaches of target NH_3_ storage to compare their influence on NO_x_ conversion and NH_3_ slip under transient conditions, all illustrated in Figure 3. Based on the results of Ref. [20] under stationary conditions, we tested percentage gradations of the determined NH_3_ breakthrough loading (black) and of the ideal NH_3_ storage curve (red). Additionally, experiments with constant NH_3_ storage levels (example in grey) were also performed. In all experiments, the catalyst was prefilled in the beginning of the test cycle. Consequently, the influence of initial catalyst loading was excluded in this study.

**Figure 4:**
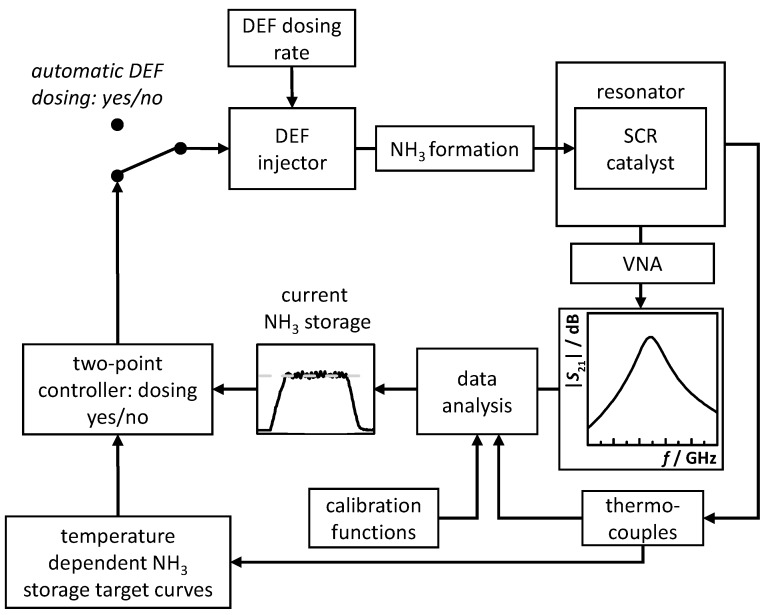
Control flow diagram of the applied RF-controlled and temperature-compensated NH_3_ storage determination and urea dosing system.

The applied control approach is illustrated in Figure 4. The DEF injector uses a defined and constant urea dosing rate, i.e., defined dosing valve open periods and valve closed periods. The formed NH_3_ adsorbs on the SCR catalyst and changes its dielectric properties. The resonance behavior of the enclosing resonator changes and the VNA acquires the corresponding resonance spectrum (complex transmission *S*_21_). The latter is used to extract the resonance parameters, and together with the catalyst temperature (determined by thermocouples) and the calibration functions of Figure 2, the current amount of stored NH_3_ is calculated. Additionally, the temperature information is used to determine the current amount of stored NH_3_ target value, which is then simply compared to the amount of stored NH_3_ on the catalyst by a two-point controller, which starts and stops the urea dosing automatically.

### 3.3. Transient Test Procedure

**Figure 5:**
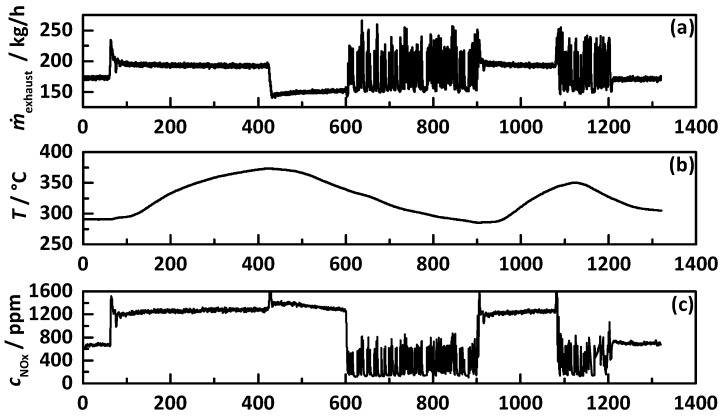

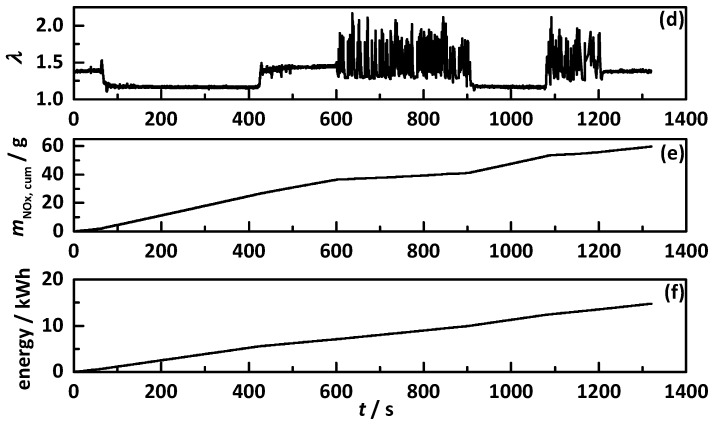
Transient test procedure with (**a**) the exhaust gas mass flow *ṁ*_exhaust_, (**b**) the catalyst temperature, (**c**) the engine out NO_x_ emissions, (**d**) the lambda signal, (**e**) the cumulative emitted NO_x_ mass, and (**f**) the consumed energy of the engine within the test cycle.

Since the available engine dynamometer setup was not able to drag the engine (fuel cut) and the adjusted exhaust pipe required medium and high loads to reach SCR active temperatures, we were not able to perform standard test cycles (NEDC, WLTC, or RDE) within this study. Instead, we used a self-defined test cycle similar to the WHSC (World Harmonized Stationary Cycle) for trucks with several jumps between different stationary operation points. The procedure of the 1320 s lasting test cycle is displayed in Figure 5, with (a) the exhaust gas mass flow *ṁ*_exhaust_, (b) the catalyst temperature, (c) the engine-out NO_x_ emissions, (d) the lambda signal, (e) the cumulative emitted NO_x_ mass, and (f) the consumed energy of the engine.

Within the test cycle, the catalyst temperature varies between 275 and 375 °C, with an exhaust mass flow of 150 up to 250 kg·h^−1^, and raw NO_x_ emissions between 120 and 1500 ppm. Additionally, the already in Ref. [20] investigated operation point with continuously changing EGR rate, mirrored in changing NO_x_ emissions and lambda (i.e., also varying humidity), was also part of the test cycle. The test procedure starts and ends with a catalyst temperature of ca. 280 °C. After a jump into full load, the catalyst heats up to the maximum temperature of ca. 375 °C within 300 s, followed by operation with medium loads and a slow cool down to ca. 275 °C. After another heat up phase in full load to ca. 350 °C, the catalyst reaches its start temperature again. Within the test cycle, the engine emits ca. 60 g NO_x_ in total and consumes energy of ca. 14.8 kWh, leading to a normalized NO_x_ emission of ca. 4000 mg/kWh.

## 4. Results and Discussion

### 4.1. Analysis Procedure

**Figure 6:**
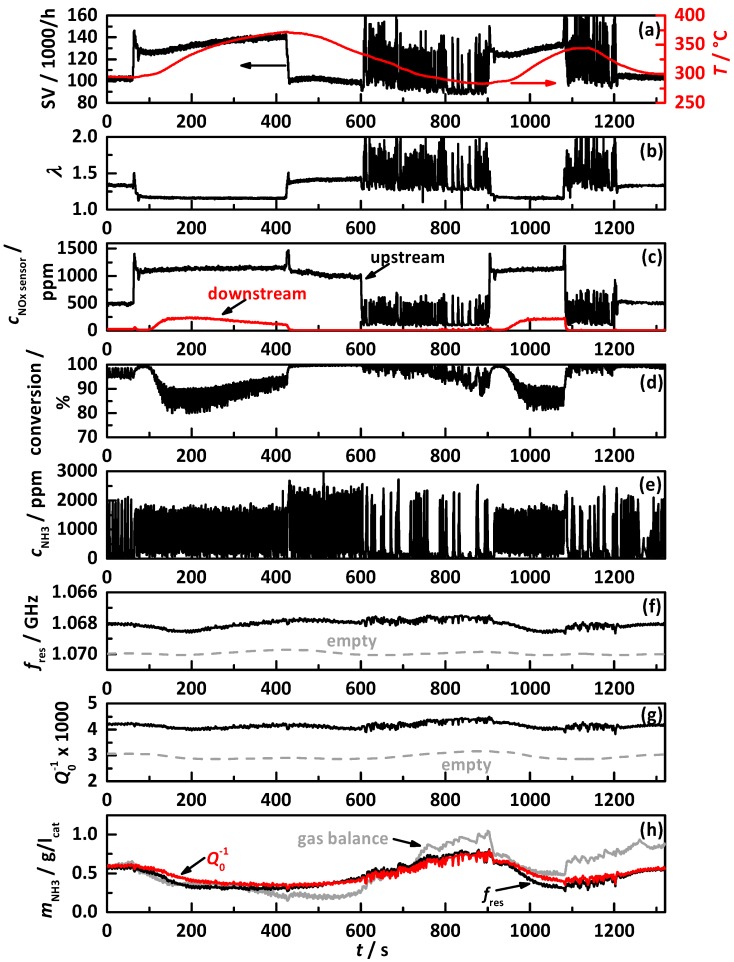
Test cycle with NH_3_ storage control on 60% of the ideal storage curve by *Q*_0_^−1^ with (**a**) the space velocity (black) and the catalyst temperature (red), (**b**) the air/fuel ratio *λ*, (**c**) the signals of the NO_x_ sensors upstream of the DEF dosing (black) and downstream of the SCR catalyst, (**d**) the calculated apparent NO_x_ conversion, (**e**) the dosed NH_3_ determined by NO_x_ sensors up- and downstream of DEF dosing, (**f**) *f*_res_ and (**g**) *Q*_0_^−1^ (black) with the calibration value for the NH_3_-free state (grey dashed), and (**h**) the amount of stored NH_3_ on the catalyst determined by *f*_res_ (black), *Q*_0_^−1^ (red, control value), and by gas balance (grey).

The analysis procedure to interpret the catalyst performance and the influence of the selected NH_3_ storage approach is explained in the following. As an example, Figure 6 shows one experimental run with an NH_3_ storage target loading of 60% of the ideal NH_3_ storage curve controlled by *Q*_0_^−1^, with (a) the space velocity (black) and the catalyst temperature (red); (b) the lambda signal of the NO_x_ sensor upstream of the DEF dosing; (c) the signals of the NO_x_ sensors upstream of the DEF dosing (black) and downstream of the catalyst (red); (d) the thereby calculated apparent NO_x_ conversion (NH_3_ slip causes a decrease in apparent NO_x_ conversion and then the latter does not represent the real NO_x_ conversion); (e) the dosed NH_3_ concentration calculated by the NO_x_ sensor signals up- and downstream of the DEF dosing; (f) *f*_res_; and (g) *Q*_0_^−1^ (black) with the calculated (by the calibration function) corresponding value for the NH_3_-free state (grey dashed); and (h) the NH_3_ loading on the catalyst determines by *f*_res_ (black), *Q*_0_^−1^ (red, control value), and calculated by gas balance (grey).

At the beginning of the test cycle with ca. 500 ppm NO_x_ emissions, the catalyst converts almost fully. By switching into full load with over 1,000 ppm NO_x_ and a space velocity of ca. 130,000 h^−1^, a short increase in conversion efficiency is visible, followed by an increase of the downstream NO_x_ sensor signal starting at second 100, which reaches its maximum of ca. 220 ppm around second 200. Until second 100, the catalyst temperature remained almost constant. So, this effect might by explained with the increased space velocity and resulting shift in reaction equilibrium, respectively, a shift of the reaction front on the catalyst surface closer to its end. With increasing catalyst temperature until second 430, a continuous decrease of downstream NO_x_ sensor signal is visible. This effect fits with the previous explanation, since the reaction front shifts more of the front of the catalyst with the thermal activated reaction rates of the SCR reactions increase. With the subsequent change to a smaller space velocity with almost the same NO_x_ emissions, the NO_x_ sensor indicates instantaneously full conversion. This behavior is also continued with further decreasing temperature after the change into the operation point, with the continuously changing EGR rate around second 600. From around second 800 on, when the catalyst temperature drops below 300 °C, first NO_x_ peaks are visible in the downstream sensor signal. The second heat-up and cool-down from second 900 on shows almost identical behavior. The dosed NH_3_ in (e) mirrors with the timely period between the dosing peaks the higher and lower required amount of NH_3_ corresponding to the current NO_x_ emission.

Within the whole test cycle, both RF signals show the almost identical course, again with the already-reported in increased uncertainty with continuously changing EGR rate Ref. [20]. The values for the NH_3_-free state are calculated by the calibration functions and the catalyst temperature. They mirror the latter. In addition, the thereby determined amount of stored NH_3_ is almost identical for both RF signals. The amount of stored NH_3_, as calculated by gas balance, suffers from increasing uncertainties especially during changing EGR rates, but generally confirms the by the RF system determined values. The NH_3_ loading within this test with control on 60% of the ideal storage curve leads to minimum NH_3_ storage of 0.3 g/l_cat_ at 375 °C and a maximum storage of 0.7 g/l_cat_. This experiment demonstrates the basic functionality of the RF approach to control the SCR catalyst on a temperature-dependent NH_3_ storage target curve.

**Figure 7:**
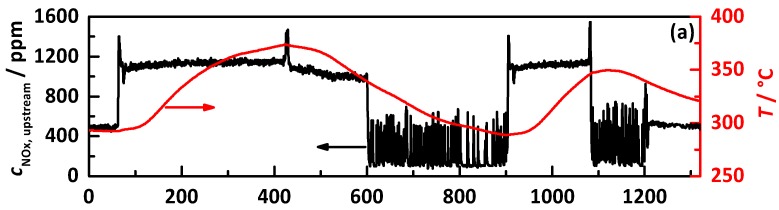

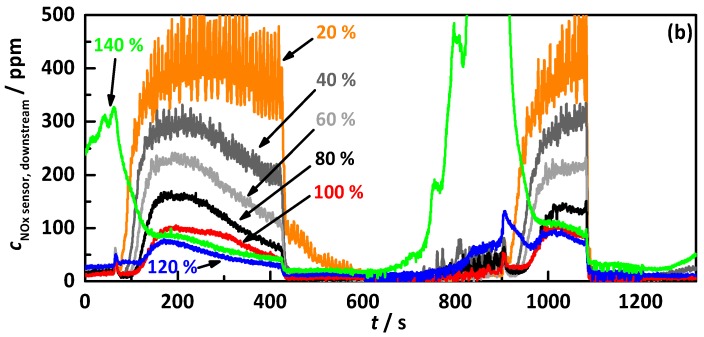
Comparison of the performed test cycles with percentage gradation of the ideal NH_3_ storage curve controlled by *Q*_0_^−1^, with (**a**) the engine out NO_x_ emissions (black) and the catalyst temperature (red) and (**b**) the signal of the downstream NO_x_ sensor for 20 to 140% of the ideal storage curve.

Since the engine setup is limited to the signals of the NO_x_ sensors without the possibility of directly differentiating between NO_x_ and NH_3_, a comparative analysis of the experiments with different gradations of the target storage curves was performed to interpret the signal of the downstream NO_x_ sensor. This is shown in Figure 7 for the experiments with the percentage gradation of the ideal NH_3_ storage curve controlled by *Q*_0_^−1^, with (a) the engine out NO_x_ emission (black) and catalyst temperature (red) and (b) the downstream NO_x_ sensor signals for a control of 20 to 140% of the ideal storage curve. It can be seen within both heat-up phases that the downstream NO_x_ sensor signal decreases with increasing NH_3_ storage until a value 120% of the ideal storage curve (blue). The signal increases again with 140% (green). The sensor signal of 20% shows, in contrast to the higher NH_3_ loadings, very noisy behavior. This can be attributed to the urea dosing pulses that occur because of the very low surface coverage. In contrast, the 140% storage (green) shows, even at the lower temperatures, a high NO_x_ sensor signal as a proof for too high NH_3_ loading and resulting high NH_3_ slip.

To also prove whether lower NH_3_ storage levels than 140% lead to NH_3_ slip, characteristic points of the test cycle were analyzed. For this purpose, the stationary conditions in the beginning of the tests cycle were used including the jump into the full load at second 60 and the change of the operation point at the highest temperature within the cycle at second 420. Due to the strong increase in space velocity at the first characteristic point, a too high NH_3_ storage, including the weaker bonded NH_3_, would be pushed out of the catalyst and detected by the NO_x_ sensor. An NH_3_ breakthrough at the second characteristic point (around second 420) at the maximum catalyst temperature would be caused by broadening and shifting of the reaction and NH_3_ storage front to the end of the catalyst. When the space velocity drops instantaneously, the reaction front moves quickly closer to the front of the catalyst. However, the remaining NH_3_ in the end would still desorb and cause NH_3_ slip that can be seen in the downstream NO_x_ sensor signal, since the temperature remains almost the same.

**Figure 8:**
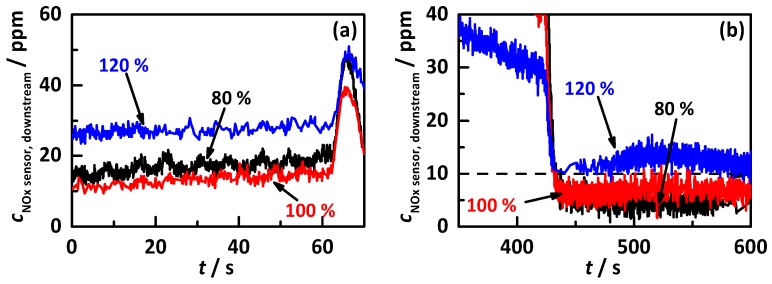
Detail view of the downstream NO_x_ sensor signals at two characteristic points of the performed test cycles with percentage gradation of the ideal storage curve for 80% (black), 100% (red) and 120% (blue), with (**a**) the first change of operation point and (**b**) the change from full into medium load at maximum catalyst temperature.

These two characteristic points of the test cycle are displayed as a detail view of the downstream NO_x_ sensor signals of Figure 7b for 80, 100, and 120% of the ideal storage curve in Figure 8, with the first characteristic point in (a) and the second in (b). It is clearly visible, for the first point, that the NO_x_ conversion increases, i.e., the sensor signal decreases from 80% (black) to 100% (red). The increase of sensor signal for 120% (blue) can be assigned easily to a constant NH_3_ slip. Still, even the control on 100% of the ideal storage curve leads to ca. 15 ppm NO_x_ downstream of the catalyst, which might be explained as the maximum possible conversion for the used conditions with high raw NO_x_ emissions and a comparable small catalyst volume, i.e., a very high space velocity of 130,000 h^−1^. A closer view on the second characteristic point shows that the signals of 80 and 100% behave almost identically with both below 10 ppm after the jump to medium load. The control on 120% leads to a higher conversion at full load but after the change of the operation point, and a clear increase in the NO_x_ sensor signal is visible and proves that 120% is already causing NH_3_ slip. 

### 4.2. Comparison of the Influence of Different NH_3_ Storage Approaches on Catalyst Performance

With the above-described procedure, all performed test cycles with control by *f*_res_ and *Q*_0_^−1^ on the storage approaches were analyzed, as illustrated in Figure 3. The following discussion is based on the apparent cumulative and normalized NO_x_ mass emission as determined by the downstream NO_x_ sensor and on the resulting apparent NO_x_ conversion efficiency within the test cycle. In addition, the experiments with NH_3_ slip were identified and the best storage level without NH_3_ slip for all three storage approaches was determined.

#### 4.2.1. Control on a Percentage Gradation of the NH_3_ Breakthrough Curve

**Figure 9:**
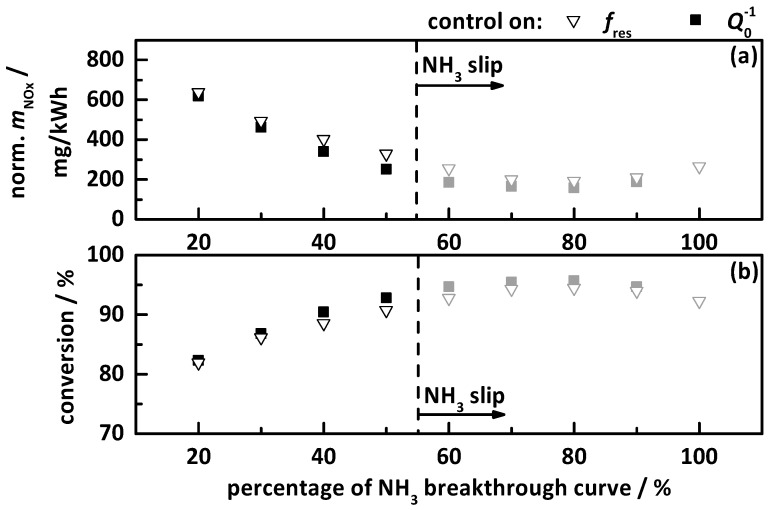
Results of the transient study for the control on percentage gradation of the NH_3_ breakthrough curve by *f*_res_ (hollow triangles) and *Q*_0_^−1^ (filled squares), with (**a**) the apparent emitted and normalized NO_x_ mass in mg/kWh as determined by the downstream NO_x_ sensor, and (**b**) the resulting apparent NO_x_ conversion within the test cycle. The storage curves right to the dashed line (grey marked) caused NH_3_ slip.

The results of the transient study for the control on a percentage gradation of the NH_3_ breakthrough curve (black curve in Figure 3) with 10% steps are displayed in Figure 9. This is for the control on *f*_res_ (hollow triangles) and on *Q*_0_^−1^ (filled squares), with (a) the apparent emitted and normalized NO_x_ mass in mg/kWh as determined by the downstream NO_x_ sensor, and (b) the thereby calculated apparent NO_x_ conversion within the test cycle. All the experiments, when NH_3_ slip was determined, are marked grey and located right to the dashed line.

In general, one can clearly see that the control on *f*_res_ and *Q*_0_^−1^ show almost identical results. A control on 20% of the NH_3_ breakthrough curve already leads to reduced NO_x_ emission of 600 mg/kWh, which corresponds to a total NO_x_ conversion of over 80%. With increasing NH_3_ storage, the emitted NO_x_ decreases continuously, until at 80% of the NH_3_ breakthrough curve, the lowest apparent NO_x_ emission of 200 mg/kWh, respectively, the highest apparent NO_x_ conversion of 95%, is reached. With further increased NH_3_ dosing, the apparent NO_x_ conversion decreases again as more and more NH_3_ slips. The analysis of the characteristic points within the cycle showed that already 60% of the breakthrough curve led to NH_3_ slip. This can be explained by the fact that the breakthrough curve only represents the storage when first breakthrough appears after the catalyst (that was empty before) was filled by a continuous NH_3_ dosing rate. In the transient tests, continuous dosing a specific storage value is applied for a longer time and NH_3_ can migrate to the end of the catalyst. This leads much earlier to a slip than applying the 100% curve. Therefore, the maximum achieved conversion efficiency without NH_3_ slip was only 90% for 50% of the breakthrough curve. With this storage control approach, the catalyst seems not to use its full potential.

#### 4.2.2. Control on a Percentage of the Ideal Storage Curve

**Figure 10:**
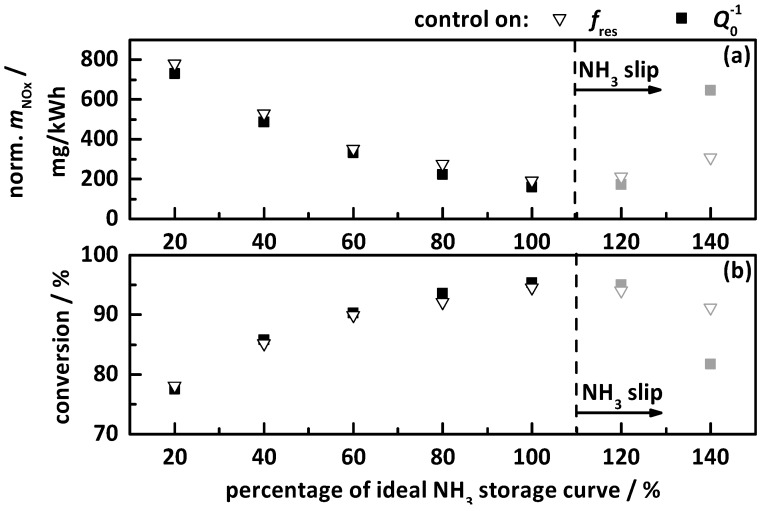
Results of the transient study for the control on percentage gradation of the ideal NH_3_ storage curve by *f*_res_ (hollow triangles) and *Q*_0_^−1^ (filled squares), with (**a**) the apparent emitted and normalized NO_x_ mass in mg/kWh as determined by the downstream NO_x_ sensor, and (**b**) the resulting apparent NO_x_ conversion within the test cycle. The storage curves right to the dashed line (grey marked) caused NH_3_ slip.

The second tested control approach was discussed in detail in the previous section in control on a percentage gradation of the ideal storage curve with 20% steps (red curve in Figure 3). The results are displayed in Figure 10 for the control on *f*_res_ (hollow triangles) and *Q*_0_^−1^ (filled squares) with (a) the apparent emitted and normalized NO_x_ mass in mg/kWh, as determined by the downstream NO_x_ sensor, and (b) the calculated apparent total NO_x_ conversion. NH_3_ slip is marked with grey and placed right of the dashed line. Again, *f*_res_ and *Q*_0_^−1^ led to almost identical results. The lowest tested storage of 20% led to NO_x_ conversion efficiency below 80%. At this point, it is important to mention that the ideal NH_3_ storage curve is always below the NH_3_ breakthrough curve (see Figure 3), which represents a lower storage for the same percentage value, also resulting in lower NO_x_ conversion due to lower NH_3_ surface coverage. As with the breakthrough curve, the conversion efficiency increases with NH_3_ storage level until a maximum at 100 and 120% is visible. With further increase to 140%, a drop in the apparent conversion efficiency can be seen. The maximum achieved apparent conversion is ca. 95%, which corresponds to ca. 200 mg/kWh. The NH_3_ slip analysis showed first slip at 120% and confirmed the in Ref. [20] under stationary conditions determined ideal NH_3_ storage curve also under transient conditions.

That this storage curve shows better results than the NH_3_ breakthrough curve might be explained by the more reduced storage at higher temperatures (compare Figure 3). Due to the better kinetics of the SCR reactions in the upper temperature range, especially with variable speeds and loads as well as non-constant raw NO_x_ emissions and space velocities, a lower surface coverage is required and too high storage yields NH_3_ slip. This demonstrates that the RF-controlled SCR system, together with the right NH_3_ storage target curve, is able to operate the catalyst with maximum NO_x_ conversion efficiency by avoiding NH_3_ slip. A bigger catalyst volume in conjunction with test cycles with more realistic conditions (e.g., lower engine loads, lower NO_x_ raw emissions, and especially more realistic space velocities) will most likely cause even better results.

#### 4.2.3. Control on a Constant Storage Value

**Figure 11:**
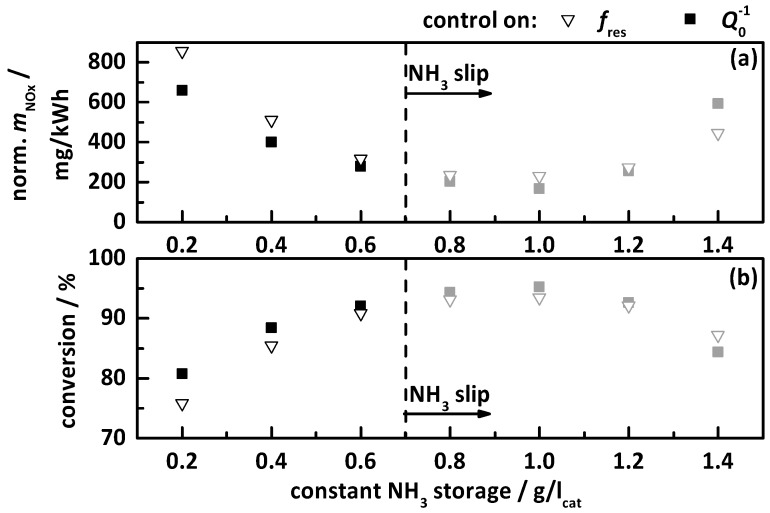
Results of the transient study for the control on a constant NH_3_ storage by *f*_res_ (hollow triangles) and *Q*_0_^−1^ (filled squares), with (**a**) the apparent emitted and normalized NO_x_ mass in mg/kWh as determined by the downstream NO_x_ sensor, and (**b**) the resulting apparent NO_x_ conversion within the test cycle. The storage curves right to the dashed line (grey marked) caused NH_3_ slip.

The last tested approach was a control on a constant storage value between 0.2 and 1.4 g/l_cat_ with steps of 0.2 g/l_cat_ (exemplarily shown as grey curve in Figure 3). The test results are displayed as analog to the breakthrough and ideal storage curves in Figure 11. There are deviations visible between the test cycles controlled on *f*_res_ and *Q*_0_^−1^, especially at highest and lowest NH_3_ storage levels. With the lowest observed constant amount of stored NH_3_ of 0.2 g/l_cat_, a maximum conversion efficiency of ca. 75 to 80% was achieved. Corresponding to the other control approaches, the apparent NO_x_ conversion efficiency based on the downstream NO_x_ sensor signal increases with NH_3_ storage, reaching its maximum at 1.0 g/l_cat_. A further increased storage level leads again to a decrease in apparent NO_x_ conversion. But based on the slip analysis, NH_3_ slip could already be the determined for 0.8 g/l_cat_, which leads to a maximum NH_3_ storage without slip of 0.6 g/l_cat_ and a NO_x_ conversion of 90%, representing 300 mg/kWh. This result fits very well to these of the experiments with the ideal storage curve. The latter provides a maximum storage of ca. 0.6g/l_cat_ at the maximum temperature of the test cycle. Once this storage value is exceeded, it causes NH_3_ slip. As already assumed, a control on a constant NH_3_ storage level for all temperatures is not capable for ideal operation of SCR catalysts controlled by the RF system.

## 5. Conclusions and Outlook

Although the RF-based state monitoring of NH_3_ storage on SCR catalysts has been in focus of research for years, large steps towards application have been made lately. Whereas in the previous works, catalyst core samples were used and the tests were conducted in gas test benches in synthetic exhausts, the lasted results have been achieved on an engine dynamometer setup in application size, and by using DEF instead of gaseous NH_3_. At first, the basic capability of this technique in real exhaust and with DEF was proven, and a first automatic RF-controlled DEF dosing on a measured NH_3_ storage degree on the catalyst surface was applied [39]. Within the next step, the temperature-dependent behavior of the RF signals was investigated and calibration functions were developed on the example of the well-studied and serial type Cu-exchanged zeolite-based catalyst Cu-SSZ-13 [20]. Besides the RF calibration, the influence of the NH_3_ storage degree on the catalyst performance was investigated as well. Nevertheless, these two studies were performed only under stationary conditions. The present paper directly continues the last-mentioned work by applying the temperature-calibrated, i.e., compensated RF system for NH_3_ storage determination on Cu-SSZ-13 under transient conditions with special focus on the influence of the NH_3_ target storage on the NO_x_ conversion efficiency and possible NH_3_ slip.

The developed calibrated RF system uses the under stationary conditions determined calibrations functions for both RF parameters *f*_res_ and *Q*_0_^−1^ in the observed temperature range of 250 to 400 °C. Together with the catalyst temperature, they were used to calculate the NH_3_ loading in real time and to control the DEF dosing valve for a desired NH_3_ storage degree. For the latter three, different storage approaches were investigated:

- the percentage gradation of the NH_3_ breakthrough storage of Ref. [20];
- the percentage gradation of the determined ideal NH_3_ storage curve of Ref. [20], i.e., the minimum required storage degree for maximum possible NO_x_ conversion, and;
- a constant temperature-independent NH_3_ storage from 0.2 to 1.4 g/l_cat_.

Due to dynamometer limitations, a self-defined test cycle similar to the WHSC was used. During this 1320 sec lasting test cycle, speed and load jump between several stationary operation points. This leads to catalyst temperatures between 275 and 375 °C, NO_x_ emissions between 120 and 1500 ppm, and very high space velocities between 90,000 up and 160,000 h^−1^. Since this study was also limited to the use of NO_x_ sensors for gas analysis with their well-known cross sensitivity to NH_3_, the entire interpretation and comparison rely on their signals. By directly comparing the downstream NO_x_ sensor signal of the experiments with stepwise increasing NH_3_ target storage, possible NH_3_ slip was identified. Therefore, the analysis of characteristic points of the test cycle was used.

The RF-controlled DEF dosing system worked very well in all performed tests for both *f*_res_ and *Q*_0_^−1^. Due to the transient conditions, the calculation of the stored NH_3_ by gas balancing is affected by small errors, but still fits well to the measured values. In the medium load operation points, full NO_x_ conversion was achieved. Within the high load operation points with very high space velocity, NO_x_ emissions and high temperatures a maximum conversion rate of ca. 95% was possible. The latter is most likely related to kinetical limitations and should be increased with adjusted catalyst volume.

The control on 50% of the NH_3_ breakthrough curve showed the best results for this approach with a maximum NO_x_ conversion of over 90% without NH_3_ slip. All higher percentages already caused NH_3_ slip. The best results of the whole study with ca. 95% NO_x_ conversion without NH_3_ slip were achieved with exactly 100% of the determined ideal NH_3_ storage curve, and confirmed the previous study [20]. The additionally observed control on a constant NH_3_ storage led to a lower maximum conversion efficiency of ca. 90% in total for a constant control on 0.6 g/l_cat_. This result fits well with the ideal storage curve, since 0.6 g/l_cat_ is exactly the highest storage of the ideal storage curve within the test cycle.

The here-presented study confirms that a directly RF-controlled SCR system on a desired NH_3_ storage on the catalyst is able to operate the latter very close to the border of NH_3_ slip with the maximum possible NO_x_ conversion efficiency. Having an application ready system may lead to a huge benefit in diesel aftertreatment control. However, in such an application, it will most likely not be used as continuous control signal, rather, it will support the existing model-based control strategies. For that purpose, an RF determined NH_3_ storage signal might be sufficient from time to time under defined conditions like constant driving for a specific time frame to be highly beneficial to the current model, as presented in Ref. [46].

The ongoing work is focusing on the implementation of the RF-SCR system in current serial application systems with special interest in field experiments on the road. Additionally, the effects of production-related influences to the catalyst substrates and their effects to system calibration and stability are still under investigation. Further interest focuses on possible poisoning effects like sulfur to the RF signal. In summary, this study was another big step towards application, with still some more to go.
